# Editorial: Catalysis rising stars in China

**DOI:** 10.3389/fchem.2023.1158203

**Published:** 2023-02-13

**Authors:** Liuqingqing Yang, Yulian He, Haifeng Xiong

**Affiliations:** ^1^ University of Michigan—Shanghai Jiao Tong University Joint Institute, Shanghai, China; ^2^ Department of Chemical Engineering, Shanghai Jiao Tong University, Shanghai, China; ^3^ State Key Laboratory of Physical Chemistry of Solid Surfaces, Collaborative Innovation Center of Chemistry for Energy Materials, College of Chemistry and Chemical Engineering, Xiamen University, Xiamen, China

**Keywords:** catalysis and biocatalysis, DFT, density functional theory, C1 catalysis, single atom catalysis, electrocatalysis

Catalysis Rising Stars in China is devoted to bringing together high-quality works from outstanding Chinese researchers in the early stages of their independent careers in order to advance our understanding of the design, synthesis, characterization, and application of catalysts. Indeed, catalysts are applied in various situations, from industrial chemical processes to biochemical reactions in the human body, to facilitate the important chemical transformation of one set of molecules into another. In this sense, catalysts play important roles in both our lives and our lifestyles. Over the decades, researchers in the catalysis field have been actively designing and synthesizing novel catalytic systems and exploring the role of catalysis in many different sectors of industry, energy, health, the environment, *etc.* Therefore, this Research Topic highlights the latest progress in the research field of catalysis, working across China.

Widely found in the natural environment and food, BaP is regulated as a Group I human carcinogen, and its degradation is of great importance. Long et al. discovered that the degradation of BaP to hydroxybenzo [a]pyrene by Mn-corrolazine can be simplified from a three-step process to one step with the aid of the oriented external electronic field *via* DFT calculations. In this way, the production of toxic epoxide intermediates in the conventional three-step process can be avoided.

Metal-support interaction (MSI) has been a research hotspot since it was first discovered in 1978. This interaction was found to be central to governing the catalytic performance of supported systems. Through systematic investigations on Cu_X_O/ZnO catalysts, Lyu et al. were able to regulate the MSI and reveal that the electron transfer from ZnO to CuO leads to the formation of an electron-rich interface, which is favorable for the adsorption of oxygen and CO oxidation reaction.

Electrocatalytic alkaline water splitting is considered an environmentally friendly technology for the production of green hydrogen. To overcome energy barriers and accelerate reaction kinetics, Li et al. have successfully developed the Ru-doped CoS_2_ catalyst for highly efficient HER. The high crystallinity of Ru-doped CoS_2_ materials was obtained with rich heterogeneous interfaces between Ru-CoS_2_ and Ru-doped MoS_2-X_. Characterization results show that this synthesized strategy could regulate the concentration of S-vacancy (CS-vacancy) precisely by the synergistic engineering of Ru doping and compositing with CoS_2_. Overall, the typical sample, which has 17.1% CS-vacancy, demonstrated the highest alkaline HER activity, with a low overpotential of 170 mV at 100 mA/cm^2^ and a TOF of 4.29 s^-1^ at −0.2 V.

Biomass holds great promise as a sustainable alternative to fossil fuel resources. Hu et al. reported an Mo, Mg co-modified Sn-β catalyst for the catalytic transformation of glucose and fructose into alkyl lactate at moderate temperatures. The results show that both the Mo species and the synergetic effect between Mo and neighboring Mg species are important in enabling the retro-aldol condensation of fructose. The selection of solvent was also found to be important for both product yield and selectivity.

The conversion of carbon dioxide (CO_2_) into value-added products is an urgent research field aimed at not only curbing carbon emissions but also realizing circular carbon economy. Zhang et al. summarize the recent metal-organic frameworks (MOFs)-based catalysts for the process of hydrogenation of CO_2_ to methanol, methane, and other C_2+_ products. The design strategies of MOF materials are also provided in order to boost the overall efficiency of CO_2_ conversion and utilization processes.

Single-atom catalysts (SACs) have attracted significant attention in the research field of heterogeneous catalysis. However, the rational design of well-performed SACs still remains challenging. Zhai et al. discuss a rational design of a Pd_1_-Pd NPs hybrid structure on a 2,6-pyridinedicarbonitrile-derived covalent triazine framework. This synthesized strategy allows for the regulation of the ratio of Pd_1_ and Pd NPs, and the typical sample presents the optimal catalytic activity with a formate formation rate of 3.66 ${mol}_{HCOOM}∙{mol}_{{Pd}^{-1}}∙h^{-1}$ .

Atomically precise gold nanoclusters (Au NCs) have highly specific surface areas and many unsaturated active sites, such that they are widely explored in the development of new heterogeneous catalysts. Hu et al. summarize the photophysical properties of several Au NCs and discuss their application in different photocatalytic reactions. The typical properties, including discrete energy levels; tunable photophysical and electrochemical properties, including visible to near-infrared absorption; microsecond long-lived excited-state lifetime; and redox chemistry are highlighted.

Methane is the cleanest fossil fuel resource with low carbon emissions; in the pursuit of carbon neutrality, catalytic methane combustion (CMC) has attracted significant attention in the efforts to curb unwanted automobile emissions as well as in green energy generation. Gao et al. focus on the transition metal oxide catalysts applied in CMC due to their comparable catalytic performance with that of precious metals. The authors also comprehensively summarize four different kinetic reaction models that have been proposed for CMC and future research prospects in this reaction field.

As mentioned, SACs catalysts constitute a research Frontier in heterogeneous catalysts. Liu et al. summarize SAC materials from the unique angle of their stability and activity at high temperatures. The authors collected the latest efforts in the development of thermally-stable SACs at elevated temperatures through the reverse-Ostwald ripening mechanism, especially for the approaches of atom trapping and vapor-phase self-assembly strategies. Other factors that can affect the thermal stability of SACs catalysts at high temperatures are also summarized, including the loading upper limit, the location of the metal single atom, the reducibility of lattice oxygen, and so on.

We are thankful to all authors for their meaningful works and to all the reviewers for their constructive suggestions and opinions. We expect that this Research Topic will encourage more researchers to discover and design novel catalysts in the future, as well as promote the in-depth development of well-performed catalytic applications.

